# Mask-mouth syndrome: a looming oral health concern

**DOI:** 10.1097/JS9.0000000000000211

**Published:** 2023-03-24

**Authors:** Chandrima Chatterjee, Bijaya Kumar Padhi, Vinay Kumar, Santosh Aryal

**Affiliations:** a Parexel International Services, New Delhi; b Department of Community Medicine and School of Public Health; c Unit of Orthodontics, Oral Health Sciences Centre, Postgraduate Institute of Medical Education and Research, Chandigarh, Punjab; d Department of Public Health, Nitte (Deemed to be University), Mangalore, Karnataka, India

*Dear Editor*,

The coronavirus disease 2019 pandemic was a public health problem that shook the healthcare systems of all nations around the world. Some other diseases have emerged collaterally with the severe acute respiratory syndrome coronavirus 2 (SARS-CoV-2) virus, namely, Mucormycosis (Black Fungus) and a much talked about oral health issue, the mask-mouth syndrome^1^,^2^. In the initial phase of the pandemic, when vaccines and drugs were under research, healthcare authorities took the nonpharmacological interventions approach to mitigate the adverse effects of coronavirus disease 2019^3^. Social distancing, lockdown, and mass sanitization were other methods adopted. The most effective way was considered to be the use of facial masks^4^. During the SARS and MERS outbreaks across the country, the masks proved to help prevent infections. These past lessons from China, Taiwan, and the Middle East have been translated worldwide. Although effective, an oral disorder has resulted from increased use of masks known as ‘mask-mouth’ (or mask-mouth syndrome). These terms had become part of the vocabulary and lives of everyone, and the ‘new normal’ had undoubtedly brought other side effects too. After prolonged periods of mask usage, dentists have encountered patients with an increased number of complaints of ‘bad breath’, ‘caries’, ‘ulcers’, or ‘bleeding gums’^5^–^8^.

In practicing the nonpharmacological interventions, a caveat was noted that these had to be followed correctly and seamlessly. Any improper usage could lead to the infection, apart from other collateral issues. The inappropriate use of masks caused headaches, acne, eye discomfort, breathlessness, impaired cognition, and mask-mouth syndrome. According to a study by Achanta *et al*.^9^, among 400 respondents, 62.3% of the respondents were women. Dry mouth (37.9%), halitosis (34.7%), and bleeding gums (2%) were the other responses reported by the study participants who wore masks for a longer time. Around 33.4% of people noticed a decrease in water intake (dehydration) after wearing the mask^1^. Another study at a university hospital evaluated self-perceived halitosis and dry mouth among adults working at or attending hospitals. The results revealed that prolonged hours of mask use increased the perception of halitosis and dry mouth. In this study, the researchers evaluated various types of masks (face masks, community masks, and medical or surgical masks) and the behavior associated with mask-mouth symptoms^7^. The user of community masks reported dry mouth and halitosis; medical/surgical masks user reported both halitosis and dry mouth; KN95-/N95-/FFP2 masks user reported dry mouth and halitosis. Furthermore, it was observed that in females, the perception of halitosis was more pronounced than in males, while it was higher in the younger age group than in people in the upper age bracket^7^. During the pandemic, when the lockdown was partially lifted or when people started to participate in their regular schedules, masks were used for extended periods to prevent SARS-CoV-2 infection. But due to these effects, people often resisted using masks, which posed a greater problem for the community. The SARS-CoV-2 virus has been shown to affect the immune system, which can lead to various alterations in responses, often resulting in bleeding and inflammation. These responses in the oral cavity manifest themselves as gingivitis and oral ulcers, further aggravated by dry mouth and other bacterial activities.

People resorted to mouth breathing, leading to dry mouth, increased the incidence of dental caries and ulcers. At this point, surface dehydration and decreased salivary flow rate (SFR) occurred, leading to oral candidiasis, halitosis, and gingivitis. These conditions are not exclusive to mask-mouth syndrome but also include immunosuppression resulting from chemotherapy or radiation therapy in cancer patients^1^.

Mask-mouth, or mask-mouth syndrome, covers many signs and symptoms such as dry mouth, dental caries, gingivitis, halitosis, and candidiasis. It is typically caused by wearing a face mask over the nose and mouth for an extended period (more than 6–8 h), resulting in a dry mouth. Furthermore, wearing a face mask increases the likelihood that the user will breathe through the mouth, resulting in surface dehydration and decreased SFR in the mouth. The decreased SFR causes problems with oral candidiasis, gingivitis, halitosis, and tooth cavities.

Dentists and oral health researchers can help people understand these signs and become aware of mask-mouth syndrome. Most members of the public who have been wearing masks for a longer period might face these problems but are unaware of how to address them. The most crucial step in preventing ‘mask-mouth syndrome’ is to maintain proper oral hygiene and eat a balanced, nutritionally balanced diet. Some preventive measures that mask users could adopt are: proper mask etiquette (changing masks after some time or after a particular procedure), changing shifts, and not working for a prolonged period that will give the person respite for removing the mask. Maintaining the moisture of the skin is another measure that can be adapted to maintain constant hydration, thus preventing acne and skin rashes. For the buccal cavity, maintaining proper oral hygiene is very important. Brushing twice and using chlorhexidine mouthwash help maintain the appropriate pH of the oral cavity, which in turn helps curb the effects of harmful bacterial and fungal activity. Diseases like candidiasis, gingivitis, or angular cheilitis can be prevented^1^,^8^.

When the pandemic was at its peak, changing masks was vital for all professions where using masks was an integral part of the job. At this point, public health practitioners become the forerunners of the awareness that is required to be spread throughout the masses. It should be kept in mind that any problems they face in maintaining oral hygiene should be addressed immediately by consulting with a dental surgeon to prevent any health problems. They can also help differentiate the symptoms of mask-mouth syndrome from those of other singular oral diseases. Any health problems, namely dental, physical, or mental, should be addressed at the earliest possible time, and professional help is always suggested instead of self-medication.

The current evidence on mask-mouth syndrome is inconclusive and needs further research to inform the population about appropriate measures. A collective understanding of the community towards a new syndrome can promote an ideal behaviors and attitudes which are essential for effective public health measures to a global crisis in the future.

## Ethical approval

No ethical approval required.

## Sources of funding

No funding received.

## Author contribution

All authors are equally contributed.

## Conflicts of interest disclosure

None.

## Research registration unique identifying number (UIN)

None.

## Guarantor

Santosh Aryal.

## Data availability statement

We have not collected any primary data for this research. The authors confirm that the data supporting the findings of this study are available within the article (and/or) its supplementary materials.

## References

[1] Bhattacharya S . ‘Mask Mouth Syndrome’ – an emerging oral health threat during the COVID-19 pandemic. J Fam Med Prim Care 2022;11:4869–4870. 10.4103/jfmpc.jfmpc_198_22 PMC9638642 36353050

[2] Muley-Itke P . ‘Mask mouth’ – a new malady affecting oral health in the COVID era. Ann Int Med Dent Res 2021:16–22.

[3] Martinelli L Kopilaš V Vidmar M . Masks during the COVID-19 pandemic: a simple protection tool with many meanings. Front Public Heal 2021;8:606635. 10.3389/fpubh.2020.606635 PMC7838459 33520918

[4] Kisielinski K Giboni P Prescher A . Is a mask that covers the mouth and nose free from undesirable side effects in everyday use and free of potential hazards? Int J Environ Res Public Health 2021;18:4344. 33923935 10.3390/ijerph18084344 PMC8072811

[5] Muley P . Mask Mouth. A Novel Threat to Oral Health in the COVID Era–Dr Pooja Muley. Dental Tribune South Asia 2020.

[6] Verma S . Mouth. Mask: Mask: a blessing or a curse for oral health. Mouth mask: A Blessing Or A Curse For Oral. http://heb-nic.in/jopd-issues/admin/freePDF/jids34p2f5ctqho2k9kd.pdf

[7] Meenakshi P Mohanan TV . Mask mouth: a novel threat to oral cavity. Res J Pharmacol Pharmacodynamics 2022;14:69–71.

[8] Kanzow P Dylla V Mahler AM . COVID-19 pandemic: effect of different face masks on self-perceived dry mouth and halitosis. Int J Environ Res Public Health [Internet] 2021;18:9180. 34501768 10.3390/ijerph18179180 PMC8431486

[9] Achanta S Sasidharan S Majji D Uppala D . ‘Mask mouth’ during COVID-19 pandemic-A myth or A truth. Int J Med Dent Res 2021;1:56–63.

